# Errors in visuospatial working memory across space and time

**DOI:** 10.1038/s41598-021-93858-6

**Published:** 2021-07-14

**Authors:** Linjing Jiang, Hoi-Chung Leung

**Affiliations:** Integrative Neuroscience Program, Department of Psychology, Stony Brook University, Stony Brook, NY 11794 USA

**Keywords:** Working memory, Network models, Human behaviour, Cognitive neuroscience

## Abstract

Visuospatial working memory (VSWM) involves cortical regions along the dorsal visual pathway, which are topographically organized with respect to the visual space. However, it remains unclear how such functional organization may constrain VSWM behavior across space and time. Here, we systematically mapped VSWM performance across the 2-dimensional (2D) space in various retention intervals in human subjects using the memory-guided and visually guided saccade tasks in two experiments. Relative to visually guided saccades, memory-guided saccades showed significant increases in unsystematic errors, or response variability, with increasing target eccentricity (3°–13° of visual angle). Unsystematic errors also increased with increasing delay (1.5–3 s, Experiment 1; 0.5–5 s, Experiment 2), while there was little or no interaction between delay and eccentricity. Continuous bump attractor modeling suggested neurophysiological and functional organization factors in the increasing unsystematic errors in VSWM across space and time. These findings indicate that: (1) VSWM representation may be limited by the functional topology of the visual pathway for the 2D space; (2) Unsystematic errors may reflect accumulated noise from memory maintenance while systematic errors may originate from non-mnemonic processes such as noisy sensorimotor transformation; (3) There may be independent mechanisms supporting the spatial and temporal processing of VSWM.

## Introduction

Visuospatial working memory (VSWM) refers to the temporary maintenance and manipulation of visuospatial information to plan and guide behaviors. Despite its central role in higher-order cognition^1^, VSWM shows varying degrees of precision. Most recent studies have highlighted the increase of recall errors in VSWM with set size^2–4^, while the effects of space and time on VSWM fidelity and the underlying neural mechanisms remain unclear. This study aimed to examine variability in VSWM performance across two-dimensional (2D) space and time, using both behavioral experiments and neurophysiology-based computational modeling.

Previous behavioral studies of humans and macaques have found that VSWM performance during a delayed response task varies across spatial locations. Both systematic error, the shift of the mean response locations, and unsystematic error, the fluctuation in responses around the mean location, have been observed. However, the behavioral pattern of these errors is inconsistent across studies, and the neural sources of these errors are unknown. Studies using an oculomotor delayed-response paradigm^5^ in macaques showed that saccades to remembered locations exhibit a systematic upward bias, with saccade endpoints systematically displaced above the target^6–8^. These studies also report that both systematic and unsystematic errors vary across space and that these errors seem to increase with increasing eccentricity^6–8^. In contrast, some studies^9^ showed a foveal bias that increases with eccentricity in human subjects, using a task requiring recalling a single remembered dot position via mouse click. Others found a quadrant bias in spatial memory recall that attracted toward the center of the quadrant in non-human primates^10^ and humans^11,12^. Thus, the exact pattern of VSWM errors across 2D space remains unclear.

One main source of VSWM variability across space is likely the functional topographic constraints of the underlying neural substrates. A distributed network of dorsal cortical regions supports VSWM processing^13–18^, most of which exhibit some form of topographic mapping of the visual space. Retinotopic maps are iconic in the early visual cortex, where nearby neurons have receptive fields at adjacent spatial locations in the visual field^19–22^. Similar implications have been made for human posterior parietal regions, including the intraparietal sulcus or lateral intraparietal area^23–26^, and prefrontal regions, including the precentral sulcus or frontal eye fields^27–31^. Since retinotopic organization of the visual system from retina to the extrastriate areas is not uniform in space, it can potentially constrain VSWM representation across the visual fields and representation in the downstream areas.

There are at least two properties of the retinotopic organization that can limit the processing and representation of visuospatial information across space, including the variation in photoreceptors and retinal ganglion cell density across the retina^32,33^ and cortical magnification of the central vision^34–41^. VSWM performances, reflected by both mean and variability of responses, may be less accurate for peripheral targets, as the cortical magnification factor decreases and the receptive field size increases with increasing target eccentricities in the primary visual cortex^38–41^. It is also uncertain to what extent the functional architecture of downstream areas may further distort VSWM representation across the visual fields. Neurons in the parietal and especially frontal regions have substantial receptive field sizes (up to 30° diameters), and these higher-order regions have much coarser functional organizations compared to the visual areas^42–46^. It remains unknown whether parietal and frontal regions exhibit an actual spatial topography other than a roughly contralateral hemispheric representation. For example, one recent study of non-human primates suggested a non-retinotopic organization in the dorsolateral prefrontal cortex that roughly divides the visual-mnemonic space into quadrants^10^.

VSWM precision not only varies across space but also changes over time. The time course of systematic and unsystematic errors in VSWM representation is not well characterized. Some studies of non-human primates found increases in unsystematic errors with increasing delay duration for up to 20 s^8,47^. In contrast, others showed that unsystematic error accumulated within the first 800 ms of the delay and stayed stable^6^. Similarly, some suggested that most systematic errors accumulated within the first second of the delay^6,8^, whereas others showed an increase in the systematic bias with delays up to 3 s^9,48–50^. One of the primary sources of VSWM errors across time may be noise accumulation over memory maintenance. Dorsolateral prefrontal recurrent microcircuitry generates self-sustained neural activity over working memory delay, potentially maintaining mnemonic contents over time^15^. Such persistent activity is characterized by attractor network models with local recurrent excitations and broad inhibitions in a topographically organized circuit^51–53^, demonstrating stochastic drifts over delay in the absence of external inputs. Drifts of the bump activities are associated with variability in behavioral errors from trial to trial over a delay^52^.

**Figure 1:**
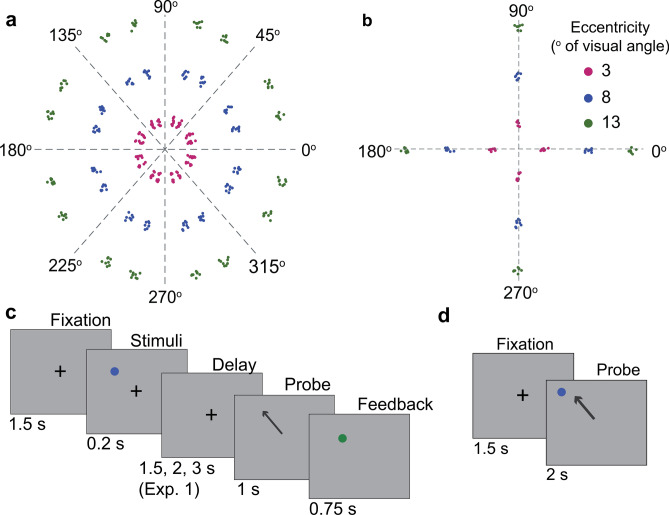
Experimental task design and stimuli. Distribution of visual stimuli across space in Experiment 1 (**a**) and Experiment 2 (**b**). Stimulus locations across trials are slightly jittered around the targets. Memory-guided saccade task (**c**) and visually guided saccade task (**d**). The arrows in (**c**) and (**d**) are shown to illustrate the memory-guided or visually guided saccade, which are not shown during the actual experiments.

Here, we investigated VSWM representation across space and time in two experiments by systematically mapping behavioral performance errors across 2D space and time in human subjects (Fig. 1). In both experiments, we recorded eye positions during a memory-guided saccade (MGS) and a visually guided saccade (VGS) task. We compared systematic and unsystematic errors of the primary and secondary saccade endpoints across different eccentricities and various lengths of memory delays and explored potential interactions between these two parameters. We expected that both systematic and unsystematic errors would increase with increasing visual eccentricity, but only unsystematic errors would increase with longer delay intervals. In addition, we applied a commonly used one-dimensional (1D), continuous bump attractor model to simulate to what extent the noise that accumulated over a delay in the recurrent microcircuitry may contribute to the observed behavioral error patterns. Neural dynamics at various eccentricities were modeled by different numbers of neurons in the model, with more neurons indexing cortical magnification at a smaller eccentricity.

## Results

### Experiment 1

#### Saccade endpoint bias and variability

**Figure 2:**
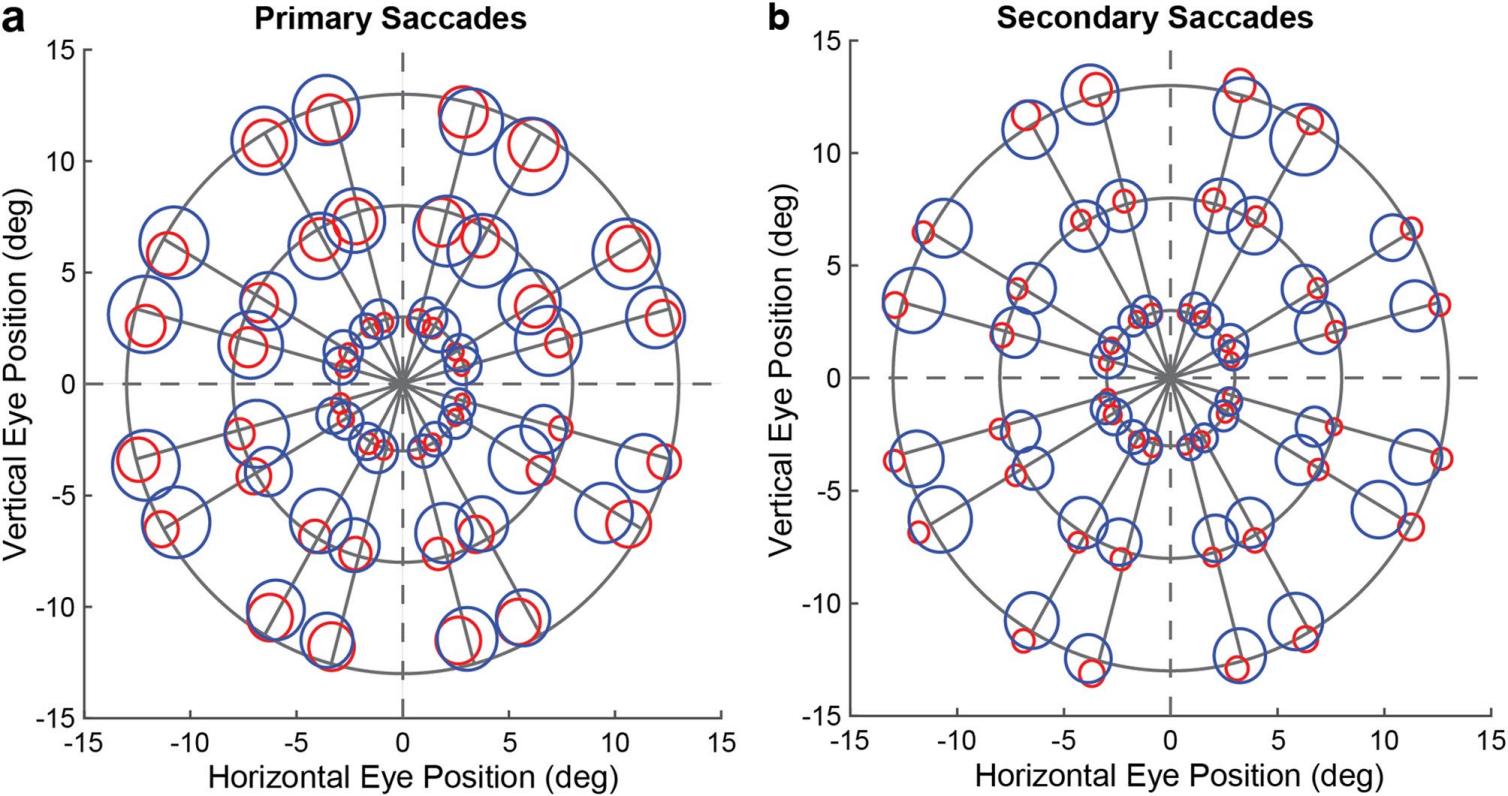
Group averages of systematic and unsystematic errors across space. The blue (MGS) and red (VGS) circles illustrate the average location of and variability in the primary saccade endpoints (**a**) and secondary saccade endpoints (**b**). Target locations are displayed as the intersection of the solid gray lines. The center of each blue/red circle represents the mean saccade endpoint (systematic errors) while the radius represents the saccade endpoint variability (unsystematic errors) averaged across subjects at each target location of Experiment 1. All data figures in the manuscript were generated in MATLAB^54^.

**Figure 3:**
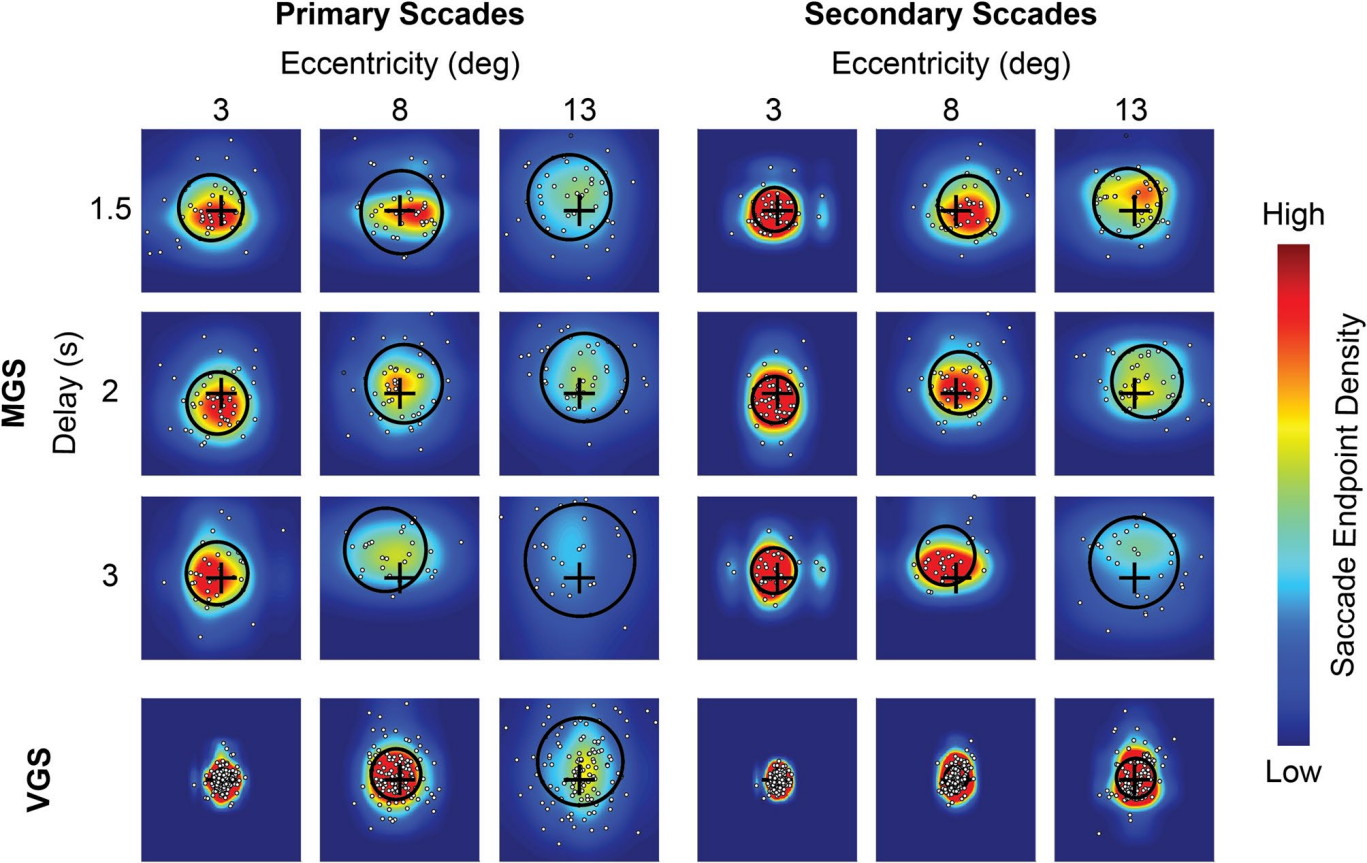
Saccade endpoint distribution across eccentricities and delays for one typical subject in Experiment 1. Heat maps display the saccade endpoint distributions that are ± 3° of visual angle around the target location for the primary and secondary saccades during the MGS and VGS tasks. The target position is marked by a black cross at the center of each heat map. The color scale indicates the increasing density of the saccade endpoints (from cool to warm colors). The small white dots mark the saccade endpoints of the individual trials. The black circle in each heat map shows the calculated systematic and unsystematic errors, with the distance between the center of the circle and the black cross representing the shift of the mean saccade endpoint position from the target (systematic error) and the radius representing the saccade endpoint variability across trials (unsystematic error).

Experimental tasks and visual stimuli are shown in Fig. 1. To visualize the overall patterns of systematic and unsystematic errors in the MGS relative to the VGS, we displayed the average variability and mean endpoints of the primary (Fig. 2a) and secondary (Fig. 2b) saccades for each target location. A systematic inward/foveal and angular bias across space was evident in the MGS (Fig. 2; Supplementary Figs. S1–S2). Compared to visually guided responses, memory-guided saccades were less accurate (*t*(9) = 4.30, *p* = .002) and displayed wider variability (*t*(9) = 7.95, *p* < .001) in endpoint positions. As expected, the secondary saccades were more accurate (*t*(9) = 3.47, *p* = .007) and less variable (*t*(9) = 13.50, *p* < .001) relative to the primary saccades, reflecting response correction. Figure 3 shows the saccade endpoint distribution across eccentricities and delays of one typical subject.

#### Effect of target eccentricity on VSWM errors

**Figure 4:**
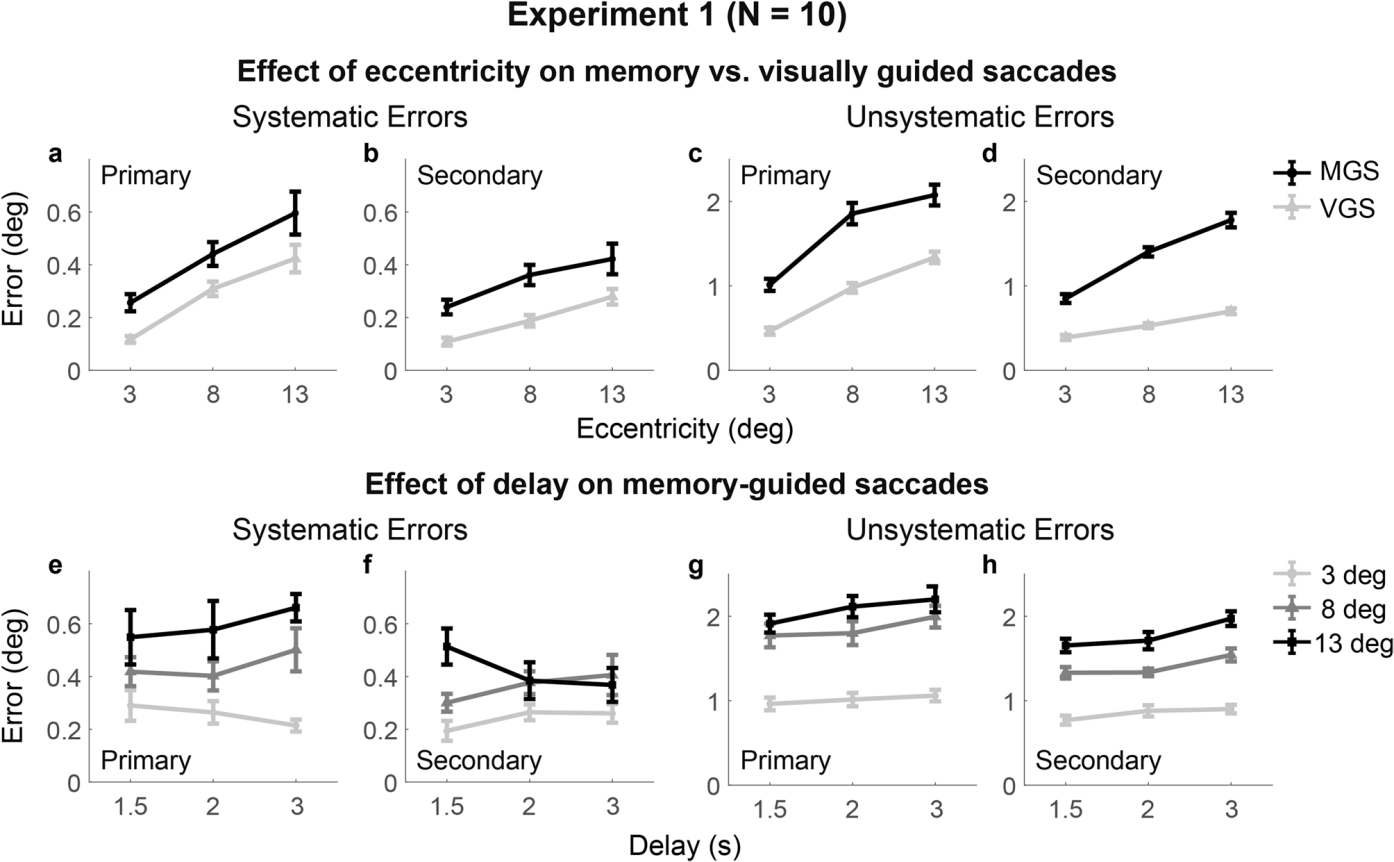
Saccade endpoint errors across tasks, eccentricities, and delays in Experiment 1. The top row shows errors in the MGS (black) and VGS (light gray) across different target eccentricities. The bottom row shows errors in the MGS across different delay intervals for each target eccentricity (3°, light gray; 8°, gray; 13°, black). Both systematic and unsystematic errors increased with increasing eccentricity, with a greater linear and quadratic increase in the MGS than in the VGS for the unsystematic errors. Unsystematic errors in the MGS accumulated from 1.5-s to 3-s delay intervals significantly for the primary and secondary saccades in a similar pattern across eccentricities.

Repeated-measures ANOVA examined the within-subject effects of eccentricity (3, 8, vs. 13° of visual angle) and task (MGS vs. VGS) (Table 1A; Fig. 4, top row). We observed significant main effects of eccentricity and task type for both systematic and unsystematic errors of the primary and secondary saccade endpoints. However, the interaction between task and eccentricity was only statistically significant for the unsystematic errors (Fig. 4c, d). Additional analyses revealed that the unsystematic errors significantly increased with increasing eccentricity linearly (primary saccades, *t* = 6.56, *p* < .001; secondary saccades, *t* = 10.22, *p* < .001) and quadratically (primary saccades, *t* = − 4.41, *p* = .003; secondary saccades, *t* = − 2.58, *p* = .059) in the MGS, but only linearly (primary saccades, *t* = 9.98, *p* < .001; secondary saccades, *t* = 13.29, *p* < .001) in the VGS. Interaction contrast analyses further confirmed a stronger linear (primary saccades, *p* > .5; secondary saccades, *t*(9) = 6.54, *p* < .001) and quadratic trend (primary saccades, *t*(9) = − 3.46, *p* = .014; secondary saccades, *p* > .1) in the MGS than the VGS for the unsystematic errors. The quadratic effect came from the smaller increase in response error from the eccentricity of 8° to 13° than from 3° to 8°. Both tasks showed similar linear trends of eccentricity for the systematic errors (*p*’s > .7; data not shown). In sum, saccade endpoint errors during the MGS showed increases in bias and variability with increasing eccentricity, with the MGS variability pattern both quantitatively and qualitatively different from the pattern of the VGS.

**Table 1:** Repeated-measures analyses of variance on VSWM errors in Experiment 1.

	Primary, systematic	Primary, unsystematic	Secondary, systematic	Secondary, unsystematic
**A. Effects of task, eccentricity, and their interaction**				
Effect of task	$F\left( {1,9} \right) = 15.0,$ $p = .004,$ $\eta _{p}^{2} = .63$	$F\left( {1,9} \right) = 109,$ $p~ < ~.001,$ $\eta _{p}^{2} = .92$	$F\left( {1,9} \right) = 14.5,$ $p = .004,$ $\eta _{p}^{2} = .62$	$F\left( {1,9} \right) = 273,$ $p~ < ~.001,$ $\eta _{p}^{2} = .96$
Effect of eccentricity	$F\left( {2,18} \right) = 22.1,$ $p~ < ~.001,$ $\eta _{p}^{2} = .71~$	$F\left( {2,18} \right) = 72.0,$ $p~ < ~.001,$ $\eta _{p}^{2} = .88$	$F\left( {2,18} \right) = 25.6,$ $p~ < ~.001,$ $\eta _{p}^{2} =$.74	$F\left( {2,18} \right) = 148,$ $p~ < ~.001,$ $\eta _{p}^{2} = .94$
Task * eccentricity	$F\left( {2,18} \right) = .104,$ $p = .90,$ $\eta _{p}^{2} = .01$	$F\left( {2,18} \right) = 3.55,$ $p = .05,$ $\eta _{p}^{2} = .28$	$F\left( {2,18} \right) = .38,$ $p = .69,$ $\eta _{p}^{2} =$.04	$F\left( {2,18} \right) = 36.8,$ $p = .000,$ $\eta _{p}^{2} = .80$
**B. Effects of delay, eccentricity and their interaction in the MGS**				
Effect of eccentricity	$F\left( {2,18} \right) = 9.34,$ $p~ = ~.002,$ $\eta _{p}^{2} = .51$	$F\left( {2,18} \right) = 38.2,$ $p~ < ~.001^{a} ,$ $\eta _{p}^{2} = .81$	$F\left( {2,18} \right) = 8.24,$ $p~ = ~.003,$ $\eta _{p}^{2} = .48$	$F\left( {2,18} \right) = 89.0,$ $p~ < ~.001,$ $\eta _{p}^{2} = .91$
Effect of delay	$F\left( {2,18} \right) = .76,$ $p = .48,$ $\eta _{p}^{2} =$.08	$F\left( {2,18} \right) = 14.1,$ $p < ~.001,$ $\eta _{p}^{2} = .61$	$F\left( {2,18} \right) = 0.02,$ $p = .98,$ $\eta _{p}^{2} =$.002	$F\left( {2,18} \right) = 34.4,$ $p~ < ~.001,$ $\eta _{p}^{2} = .79$
Eccentricity* delay	$F\left( {4,36} \right) = 1.03,$ $p = .41,$ $\eta _{p}^{2} =$.10	$F\left( {4,36} \right) = 1.49,$ $p = .25^{a} ,$ $\eta _{p}^{2} = .14$	$F\left( {4,36} \right) = 4.61,$ $p = .004,$ $\eta _{p}^{2} =$.34	$F\left( {4,36} \right) = 3.17,$ $p = .052,$ $\eta _{p}^{2} = .26$

“a” indicates p-value corrected by the Greenhouse–Geisser method due to violation of sphericity.

#### Effect of delay on VSWM errors across eccentricity

To examine how VSWM errors varied across delay durations at the different eccentricities, we performed a two-way repeated-measures ANOVA with eccentricity (3, 8, vs. 13° of visual angle) and delay (1.5, 2 vs. 3 s) as within-subject factors. We found a significant main effect of delay in unsystematic errors but not systematic errors (Table 1B; Fig. 4, bottom row). Specifically, unsystematic errors during the MGS increased with delay durations linearly for both the primary and secondary saccades (respectively: *t*(9) = 4.82, *p* = .002; *t*(9) = 7.36, *p* < .001), showing significant increases from the 1.5-s to 3-s delay conditions (primary saccades, *t*(9) = 4.82, *p* = .003; secondary saccades, *t*(9) = 7.36, *p* < .001) and from the 2-s to 3-s delay conditions (primary saccades, *t*(9) = 3.17, *p* = .034; secondary saccades, *t*(9) = 6.13, *p* < .001).

However, there were no significant interactions between eccentricity and delay in the MGS, except for the systematic errors of the secondary saccades (Fig. 4f). The interaction effect was related to a linear decrease of systematic errors with increasing delay at the largest eccentricity (13° of visual angle) as opposed to a linear increase at the mid eccentricity (8° of visual angle) (linearity contrast: *t*(9) = − 3.53, *p* = .039), though both linear trends were not statistically significant (8° of visual angle: *t* = 1.64, *p* = .27; 13° of visual angle: *t* = − 2.10, *p* = .13). The delay by eccentricity interaction was approaching significance for the unsystematic errors in the secondary saccades.

### Experiment 2

**Figure 5:**
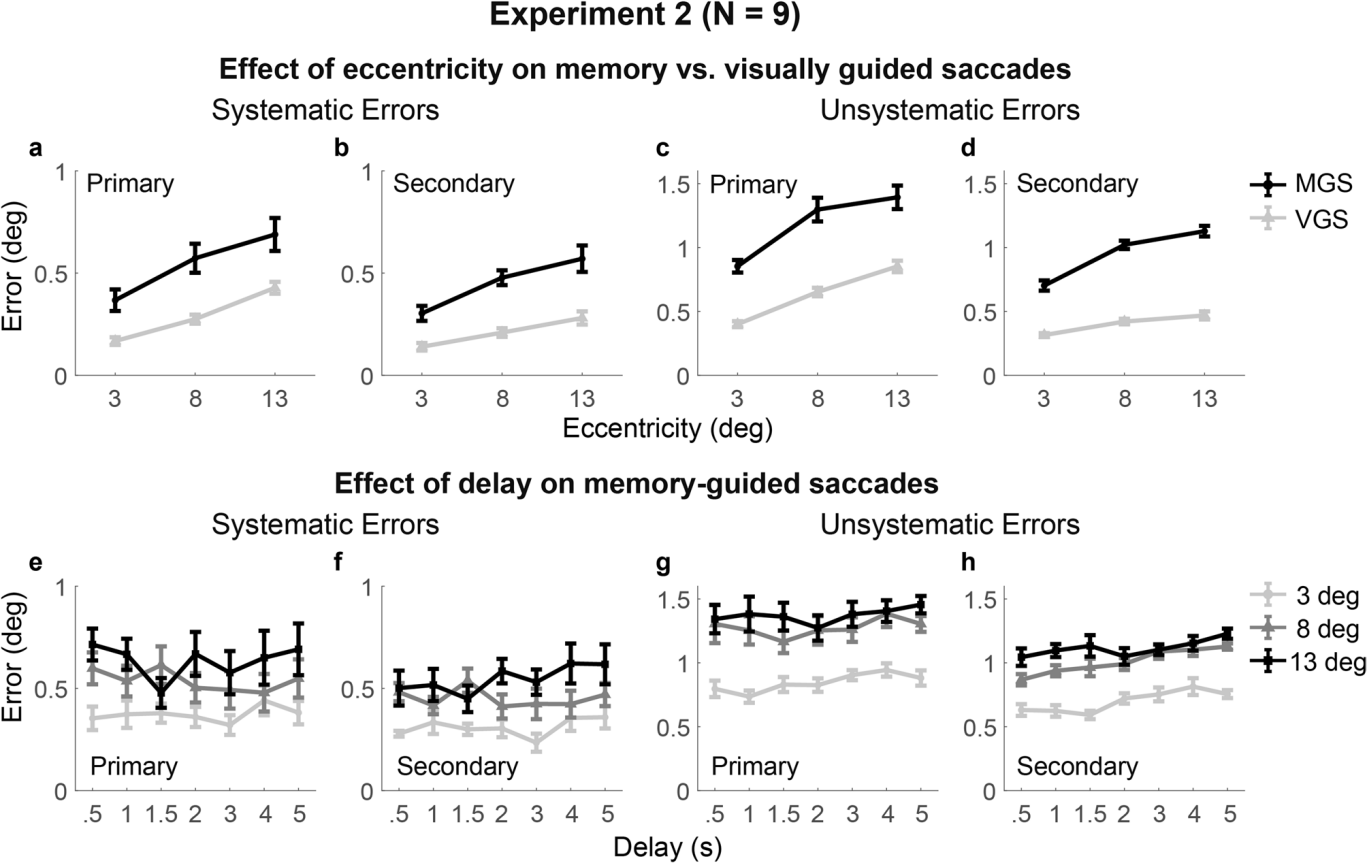
Saccade endpoint errors across tasks, eccentricities, and delays in Experiment 2. See notations in Fig. 4. Systematic and unsystematic errors increased with increasing eccentricity, with a greater linear and quadratic increase in the unsystematic errors in the MGS than the VGS. Unsystematic errors in the MGS accumulated from the 0.5-s to 5-s delay intervals significantly for the secondary saccades and were trending significance for the primary saccades in a similar pattern across eccentricities.

In Experiment 2, we aimed to replicate the results of Experiment 1 and further investigate the effect of retention duration on VSWM performance. We thus examined response errors across a wider range of delay intervals (0.5, 1, 1.5, 2, 3, 4, and 5 s). Again, repeated-measures ANOVAs (Table 2A; Fig. 5, top row) revealed significant main effects of task type and eccentricity for all types of errors and a significant interaction between task and eccentricity for the unsystematic errors of the secondary saccades. During the MGS, the unsystematic errors of the secondary saccades increased with increasing eccentricity both linearly (*t*(8) = 15.23, *p* < .001) and quadratically (*t*(8) =  −3.98, *p* = .008); such linear and quadratic increase was significantly greater in the MGS than the VGS (linear contrast: *t*(8) = 7.36, *p* < .001; quadratic contrast: *t*(8) =  −3.10, *p* = .030). Other types of errors only linearly increased with increasing eccentricity (p’s < .003, data not shown) without significant quadratic trends (p’s > .10, data not shown).

**Table 2:** Repeated-measures analyses of variance on VSWM errors in Experiment 2.

	Primary, systematic	Primary, unsystematic	Secondary, systemic	Secondary, unsystematic
**A. Effects of task, eccentricity, and their interaction**				
Effect of task	$F\left( {1,8} \right) = 14.74,$ $p = .005,$ $\eta _{p}^{2} = .65$	$F\left( {1,8} \right) = 130.62,$ $p < ~.001,$ $\eta _{p}^{2} = .94$	$F\left( {1,8} \right) = 21.81,$ $p = .002,$ $\eta _{p}^{2} = .73$	$F\left( {1,8} \right) = 385.8,$ $p < ~.001,$ $\eta _{p}^{2} = .98$
Effect of eccentricity	$F\left( {2,16} \right) = 16.99,$ $p~ < ~.001,$ $\eta _{p}^{2} = .68~$	$F\left( {2,16} \right) = 40.43,$ $p~ < ~.001,$ $\eta _{p}^{2} = .83~$	$F\left( {2,16} \right) = 30.07,$ $p~ < ~.001,$ $\eta _{p}^{2} = .79~$	$F\left( {2,16} \right) = 154.3,$ $p~ < ~.001,$ $\eta _{p}^{2} = .95~$
Task * eccentricity	$F\left( {2,16} \right) = 0.41,$ $p = .62,$ $\eta _{p}^{2} = .048$	$F\left( {2,16} \right) = 2.60,$ $p = .14^{a} ,$ $\eta _{p}^{2} = .25$	$F\left( {2,16} \right) = 1.58,$ $p = .24,$ $\eta _{p}^{2} = .17$	$F\left( {2,16} \right) = 38.72,$ $p < ~.001,$ $\eta _{p}^{2} = .83$
**B. Effects of delay, and the interaction between eccentricity and delay in the MGS**				
Effect of eccentricity	$F\left( {2,16} \right) = 8.31,$ $p~ = ~.003,$ $\eta _{p}^{2} = .51$	$F\left( {2,16} \right) = 19.58,$ $p~ < ~.001,$ $\eta _{p}^{2} = .71$	$F\left( {2,16} \right) = 15.36,$ $p~ < ~.001,$ $\eta _{p}^{2} = .66$	$F\left( {2,16} \right) = 125.86,$ $p~ < ~.001,$ $\eta _{p}^{2} = .94$
Effect of delay	$F\left( {6,48} \right) = 0.82,$ $p~ = ~.56,$ $\eta _{p}^{2} = .09$	$F\left( {6,48} \right) = ~2.05,$ $p~ = ~.077,$ $\eta _{p}^{2} = .20$	$F\left( {6,48} \right) = 0.87,$ $p~ = ~.53,$ $\eta _{p}^{2} = .10$	$F\left( {6,48} \right) = 6.68,$ $p~ < ~.001,$ $\eta _{p}^{2} = .46$
Eccentricity * delay	$F\left( {12,96} \right) = 1.73,$ $p~ = ~.071,$ $\eta _{p}^{2} = .18$	$F\left( {12,96} \right) = 0.66,$ $p~ = ~.78,$ $\eta _{p}^{2} = .08$	$F\left( {12,96} \right) = 1.42,$ $p~ = ~.17,$ $\eta _{p}^{2} = .15$	$F\left( {12,96} \right) = 1.29,$ $p~ = .24,$ $\eta _{p}^{2} = .14$

“a” indicates p-value corrected by the Greenhouse–Geisser method due to violation of sphericity.

The effects of delay on VSWM performance (Table 2B; Fig. 5, bottom row) were similar to those reported in Experiment 1. The main effect of delay on the unsystematic errors was statistically significant for the secondary saccades and approaching significance for the primary saccades but did not show significant interactions with eccentricity. Post-hoc comparisons revealed significant differences in the unsystematic errors between the 4-s and 0.5-s delay (*t*(8) = 6.08, *p* = .006) and between the 4-s and 1.5-s delay (*t*(8) = 4.90, *p* = .025) for the secondary saccades. There were no significant effects of delay or delay by eccentricity interactions on the systematic errors.

We performed additional analyses to further study the effects of delay and eccentricity on memory-guided saccades. First, we collected a replication dataset for Experiment 2 from six previous subjects to examine if performance stabilized after extensive practice. Repeated-measures ANOVAs revealed similar effects of eccentricity and delay in this replication dataset (Supplementary Fig. S3; Table S1) to those observed in Experiment 1 and in the first dataset of Experiment 2. Notably, unsystematic errors increased significantly and linearly with longer delay durations for both the primary (*t*(30) = 4.20, *p* = .001) and secondary (*t*(30) = 5.62, *p* < .001) saccades as in Experiment 1.

Second, we conducted analyses at the individual level by regressing both types of the MGS errors on eccentricity, delay duration, and their product for each of the nine participants (Supplementary Fig. S4; Table S2). The coefficient of eccentricity for the systematic error was significantly greater than zero in the primary and secondary saccades of two subjects, though it was greater than zero for the unsystematic errors in six subjects’ primary saccades and eight subjects’ secondary saccades. The 95% confidence intervals (CI) of delay did not include zero for the unsystematic errors, except for one subject’s primary saccades and three subjects’ secondary saccades. All 95% CI of delay for the systematic errors and most 95% CI of the product of eccentricity and delay did not include zero. These individual results confirmed the group analysis: in the MGS, unsystematic errors increased with larger target eccentricities and longer delay durations, though the effects of delay were weaker than that of eccentricity.

Taken together, in Experiment 2, saccade endpoint errors in the MGS also showed a greater increase of variability with increasing eccentricity (3 to 13° of visual angle) and delay duration (0.5 to 5 s) than in the VGS, and the two factors seem to act independently from each other. By contrast, the systematic bias did not vary across delay durations and increased across eccentricities similarly in the MGS and VGS.

### Simulated outcome for VSWM across space and time

Lastly, we examined the potential neurophysiological sources of delay and eccentricity effects on VSWM errors. Applying a 1D continuous bump attractor model^52^, we simulated the mean and variability of the MGS endpoints across seven delays (0.5 to 5 s) and three eccentricities (3 to 13° of visual angle) as used in Experiment 2. The number of neural units in the model indexed cortical magnification across the visual field (3°, 933 neurons; 8°, 402 neurons; 13°, 256 neurons), based on the cortical magnification factor estimated in a human retinotopic mapping study^55^. We simulated 10,000 trials for each combination of delay and eccentricity (Supplementary Fig. S5). As expected from previous modeling^51^, while the simulated systematic errors did not show considerable variation across eccentricity or delay, the simulated unsystematic errors showed substantial increases across both eccentricity and delay. In sum, the simulated data replicated the main effects of delay, but failed to capture the systematic error patterns and the independent effects of eccentricity and delay on the MGS, as shown in the behavioral data.

## Discussion

In the present study, we investigated how VSWM representation varies across 2D space and time. Our behavioral data showed that both systematic and unsystematic errors of memory-guided saccades increased with eccentricities. However, only unsystematic errors showed a qualitatively different pattern from visually guided saccades. These findings follow the cortical magnification model, indicating that the retinotopic organizations of the visual pathway may constrain VSWM representations. It is also evident that unsystematic errors of memory-guided saccades increased with the retention interval for up to 5 s, albeit the effect seemed to be less robust and more variable across subjects than the effect of eccentricity. We postulate that unsystematic errors are associated with accumulated neuronal noise in memory maintenance in accordance with neurophysiology models, whereas systematic errors may originate from non-memory-based processes such as sensorimotor transformation. The lack of eccentricity by delay interaction in the MGS further suggests independent mechanisms underlying spatial and temporal processing of VSWM.

Our experiments consistently showed increased systematic and unsystematic errors in memory-guided saccades with increasing target eccentricity. These findings closely corroborate with previous studies reporting greater response errors with larger target eccentricity in macaques using a similar MGS task^7^ and in humans using a task requiring manually controlled mouse clicks to locate targets in space^9^. Extending previous research, we further show that the variability of memory-guided responses across eccentricity was beyond a quantitative difference from visually guided responses. In particular, unsystematic errors in the MGS increased linearly and quadratically with increasing eccentricity compared to a smaller linear increase in error in the VGS. These findings suggest that VSWM representations are not homogeneous across the 2D space and are nonlinear across eccentricities. VSWM representations may have a sharper transition from fovea (< ~ 3°) to parafovea (< ~ 5°) and demonstrate similar properties beyond parafovea (> ~ 5°)^32,33,56^.

A possible neural mechanism underlying the spatial heterogeneity of VSWM errors is the structure of functional topographical mapping of space in the visual system. Retinotopic properties of the dorsal visual pathway, such as cortical magnification, may constrain VSWM representations across the visual field similarly to constraining visual perceptual and attentional performances across space^36,39,57–59^. Intriguingly, previous studies of visual short-term memory have shown a decrease in visual working memory capacity for letters with increasing eccentricity from 4° to 10° of visual angle, and the effect was partially attenuated after rescaling the stimulus in accordance with the cortical magnification factor^60^. Our findings of a nonlinear increase in unsystematic errors across eccentricity further extend the cortical magnification hypothesis to VSWM for spatial locations. As shown in previous studies of perceptual acuity^39^, the cortical magnification factor is also a nonlinear function of eccentricity, which shows a gradually slower decrease from the eccentricity of 1.5° to 12° of visual angle. The greater VSWM errors for peripheral targets may stem from spatial heterogeneity of perceptual processing in early visual regions. Alternatively, it could stem from an uneven mnemonic representation across space in the lower-level visual regions^16,17^ and in the higher-level posterior parietal and frontal regions with further distortion in their retinotopic organization^10,44^. Thus, cortical magnification of the early visual regions may limit both perceptual encoding and mnemonic maintenance of spatial information in the peripheral visual field.

How exactly may cortical magnification impact VSWM precision across space? There are at least two mechanisms, including a decreased cortical magnification factor and an increased receptive field size as the target eccentricity increases^38,40,41,61,62^. Since cell density in the central visual representation of V1 is roughly the same^63^ as or even higher^64^ than that in the peripheral representation, decreases in cortical magnification factor with eccentricity means more neurons dedicated to foveal processing. As illustrated in the previous modeling studies^51^ and in our simulation data (Supplementary Fig. S5), a smaller number of neurons in a local VSWM microcircuit would increase the stochastic drift in the population activity and hence increase the variance of the network output. It is possible that the larger unsystematic error with increasing eccentricity in our MGS data is the behavioral manifestation of the cortical magnification factor and variability in recurrent network activity across cortical space. In addition, larger receptive field size may also reduce the quality of VSWM representation in the periphery, as a broad spatial tuning may reduce the precision of both encoding and maintenance of VSWM. Future studies could further examine how the size of the memory field in the bump attractor network affects VSWM errors by systematically varying the structure and strength of the excitatory and inhibitory synaptic transmission.

The second important observation is the different time course of systematic and unsystematic errors over the delay. We found an accumulation of unsystematic errors in memory-guided saccades as the delay period extended for up to 5 s, whereas a more randomly distributed systematic error over time. It aligns with many previous studies of working memory, showing that the longer the retention interval, the greater the variability of memory recall for color^65,66^, orientation^48,66,67^, face stimuli^66^, and spatial location^8,47^. In contrast, the mean recall error for spatial targets does not vary significantly with the delay duration^6,8,47^. In particular, our results replicated and extended the spatial working memory study by White and colleagues^8^, who used a similar MGS paradigm in macaque monkeys. However, compared to patterns of unsystematic error across the 2D space, delay-related error patterns seemed to show greater individual differences and seemed independent of eccentricity. Nonetheless, the effect of delay was stable across multiple sessions and reproduced in the replication dataset (Supplementary Fig. S3).

What is the neural substrate of the degraded memory precision over time, as reflected by the increased recall variability with longer delay durations? The representation of VSWM contents or goals is associated with sustained neural activity observed in distributed cortical and subcortical regions during the memory delay^15^, and variability of persistent neural activity during the delay may underlie the behavioral response variability in individual trials^5,52,68,69^. This neural variability gradually accumulates over time, associated with the degradation of memory representation with increased retention interval^52^. As modeled by previous studies^51^ and the present study (Supplementary Fig. S5), in a bump attractor model, random drifts of the population neural activity enhance over time in the absence of external sensory inputs, increasing unsystematic, not systematic errors with longer delay durations. Beyond the local recurrent microcircuitry mechanisms, other neural mechanisms, such as short-term synaptic plasticity^70–72^, may also account for changes in memory precision over the delay.

Whereas unsystematic errors may stem from memory-related processes, systematic errors seem less dependent on memory maintenance. Here, we found that systematic errors did not accumulate over the delay intervals in our behavioral findings and computational simulation. Moreover, systematic errors have been shown to vary with the brightness of the room^8^, initial head and eye positions^6^, and post-saccadic feedback^7^. Thus, systematic errors may rely on a non-memory-based mechanism, such as a noisy sensorimotor transformation process that inaccurately translates retinotopic signals into the motor commands^6^. In particular, one study^73^ showed that discharging activities of the collicular saccade-related burst neurons are similar in an accurate visually guided saccade and an inaccurate memory-guided saccade, indicating that systematic errors may arise from addition or omission of signals that are downstream from the superior colliculus. These signals may relate to kinematic constraints on saccade generation^74^, such as a compensation of presaccadic orbital position, but the exact role of such signals in generating systematic errors in memory-guided responses remains unclear. Nevertheless, we could not rule out that systematic errors originate from other cortical regions involved in spatial processing and sensorimotor transformation such as FEF^75^ and IPS^76,77^. For instance, FEF has been shown to demonstrate a quadrant-wise neural organization, possibly related to a systematic bias of memory responses toward the center of the quadrant^10^.

Though our main findings support a memory-based theory of unsystematic errors and a non-memory-based theory of systematic errors, it is important to note other possibilities. Sensorimotor transformations may also play a role in the accumulation of response variability over time during the early delay^6^ or throughout the delay period^75^. It is also unknown whether systematic errors may relate to memory maintenance in some situations. Systematic errors may substantially increase after 5 s, the longest delay interval we used. Moreover, it has been shown that the mean recall errors for orientation^48,49^ or color^50^ increased over memory delays when multiple items were presented and when the items were actively maintained in memory^50^. More studies are needed to examine where, when, and how systematic and unsystematic errors occur. Spatial working memory paradigms that do not rely on sensorimotor transformation, such as a delayed match-to-sample task^78^ may be a good alternative to test the dependence of behavioral errors on memory maintenance. Examining systematic and unsystematic errors of VSWM in clinical models such as Parkinson’s disease^79^ and schizophrenia^80^ may also further the understanding of the nature of the behavioral errors and could potentially help develop behavioral markers to aid the diagnosis of these disorders.

Another intriguing finding in the present study is the difference between primary and secondary saccades. Overall, secondary saccades showed a more robust task by eccentricity interaction and effect of delay compared to primary saccades. It is possible that primary and secondary saccades reflect different components of VSWM. Primary saccades may be more closely linked to an initial motor plan, whereas corrective saccades may more closely reflect the quality of the VSWM representation^81–83^. As such, errors in the primary memory-guided saccades may involve motor errors that are not differentiable from the visually guided responses, explaining the weaker interaction between task and eccentricity. Consistently, a smaller delay effect on the primary saccades may indicate constant motor errors across delays.

Finally, we found little or no interaction between eccentricity and delay, suggesting that the spatial and temporal processing of VSWM may be largely independent. It is possible that VSWM maintenance across time relates to local neural dynamics, such as the stochastic drifts of the neural activity simulated by the bump attractor model. In contrast, VSWM representation across space is probably constrained by a more global neural mechanism, such as the long-range, topographical mapping within and across cortical regions. Such contrast between the local and global mechanisms may explain the invariance of systematic errors across delays and eccentricity by delay interactions in the simulated data (Supplementary Fig. S5) since the model only captures local neural dynamics but not changes in inter-regional and intra-regional topography. Future models should incorporate both local and global mechanisms to simulate spatial heterogeneity in VSWM processing. For instance, building a two-dimensional VSWM model that incorporates the intra-regional (non-)topographical organization along the dorsal visual pathway measured by fMRI^22^ or electrophysiology^10^ would be crucial to understand VSWM processing across space. Moreover, large-scale circuit models can be further integrated with topographic factors, such as inter-regional topographical connections^84^ and cortico-cortical sampling^85^, to capture spatial processing in VSWM better.

In summary, the present study demonstrates how saccade endpoint errors vary across two-dimensional space and time using an oculomotor delayed-response task in human subjects. We observed different time courses of systematic and unsystematic error over the retention interval, during which the systematic errors fluctuated randomly, whereas the unsystematic errors appeared to accumulate for up to 5 s. We propose that unsystematic errors, not systematic errors, reflect more on memory-related processes. We also found an increase in systematic and unsystematic errors with increasing target eccentricity. The interaction between eccentricity and task was significant for unsystematic errors, indicating a potential constraint of the retinotopic organization on VSWM representation. Finally, the lack of interaction between eccentricity and delay suggests potentially distinct mechanisms of VSWM processing across space and over time.

## Methods

### Participants

Ten healthy individuals from Stony Brook University (3 females, median age of 19.5 years, range from 18 to 24 years) participated in Experiment 1. Nine healthy individuals (one is the author), five females (median age of 22 years, range from 20 to 26 years) participated in Experiment 2. All individuals had normal or corrected-to-normal vision and had no history of neurological or psychiatric disorders by self-report. All participants gave written informed consent for the study. The study was conducted according to procedures approved by the Institutional Review Board of the Office of Research Compliance at Stony Brook University. All methods were carried out in accordance with relevant guidelines and regulations.

### Eye-tracking and visual stimuli

Participants performed the task in a bright room 50 cm from a ViewSonic CRT monitor (36.4 cm × 27.5 cm, 85 Hz refresh rate) with their head stabilized by a chin rest. Binocular eye position was recorded by a head-mounted eye tracker (Eyelink II, SR Research, Ottawa) at 500 Hz in pupil-only mode. A 13-point calibration was performed at the beginning and in the middle of the experiment to make sure eye positions are correctly registered. We performed a manual drift correction procedure at the beginning of each trial. Trials where subjects’ eye positions shifted 2° away from the center fixation during the stimulus and the probe period were discarded and repeated in a random order later in the block.

We used Experiment Builder software (SR Research, Ottawa) to present visual stimuli throughout the experiment. A black cross that subtended 0.8° of visual angle was used for fixation. A blue dot that subtended 0.8° of visual angle was used for the target but turned into green color during the feedback phase. In Experiment 1, there were 3 target eccentricities (3°, 8°, 13° of visual angle) and 16 target polar angles (from 15° to 345°), leading to 48 possible target locations (Fig. 1a). In Experiment 2, there were 3 target eccentricities (3°, 8°, 13° of visual angle) and 4 target polar angles (0°, 90°, 180°, 270°), leading to 12 possible target locations (Fig. 1b). We also jittered the stimulus positions from trial to trial to minimize potential long-term memory effects. Specifically, the stimulus positions were sampled from a bivariate uniform distribution centered 0.5° of visual angle around a primary target eccentricity and 2.5° around its polar angle. To account for the stimulus jittering in the subsequent analysis, we linearly translated saccade endpoint positions from trial to trial in the 2D pixel space such that their corresponding target locations were aligned by eccentricity and polar angle.

### Task design and procedure

#### Visually guided saccade (VGS) task

Participants first maintained fixation for 1–2 s on a cross in the center of the screen. The cross then disappeared, followed by the display of a target dot for 2 s. The participants were instructed to shift their gaze to the target as quickly and as precisely as possible when it appears (Fig. 1d).

#### Memory-guided saccade (MGS) task

Similar to the VGS task, except the target dot appeared for a duration of 200 ms followed by a variable delay interval (Experiment 1: 1.5, 2, or 3 s; Experiment 2: 0.5, 1, 1.5, 2, 3, 4, or 5 s) before the required saccadic response. Participants were asked to remember the target location and maintain fixation at the center cross during the delay. When the fixation cross disappeared at the end of the delay period, participants shifted their gaze to the remembered location of the target. One second after the fixation cross offset, the target dot reappeared in green for 750 ms to serve as a feedback signal, and the participant shifted their gaze to the feedback dot before looking back at the center fixation for the subsequent trial (Fig. 1c).

#### Procedure

In Experiment 1, The same group of 10 participants performed the MGS and VGS tasks in two sessions on two separate days. We counterbalanced the order of the two tasks across participants. Each session consists of a practice block with 24 trials and 8 experimental blocks with 48 trials per block, resulting in 408 trials. For the MGS task, the delay duration of each trial was pseudo-randomly selected from all 3 possible durations for each eccentricity. The target location of each trial was pseudo-randomly selected from all 48 possible locations. Within a block, all 48 possible target locations appeared with no repetition so that each location appeared 8 times throughout the entire task.

Experiment 2 was similar to Experiment 1, except seven different delay intervals (0.5, 1, 1.5, 2, 3, 4, 5 s) were used in the MGS task to better characterize the MGS errors across time. During the main data collection (Table 2; Fig. 5), 9 participants performed the MGS and VGS tasks in two sessions on two separate days. Moreover, we collected a replication dataset (Supplementary Table S1; Fig. S3) from 6 of the 9 participants who performed the MGS and VGS sessions again on two separate days. Both the MGS and VGS sessions consisted of 14 experimental blocks with 30 trials per block resulting in 420 trials. The target location of each trial was pseudo-randomly selected from all 12 possible locations, and the delay duration was pseudo-randomly selected from all 7 possible intervals. Trials were pseudo-randomly divided up among the blocks with experimental conditions (locations and delay durations) interleaved in each block. There were no repetitive target locations (either eccentricity or polar angle) or delay intervals for consecutive trials.

### Saccade data analysis

We first imported raw eye-position data to DataViewer (SR Research, Ottawa) for preprocessing. Although binocular data was collected for most subjects, only right eye-position was used for the final data analysis, except for one subject in Experiment 1 and one subject in Experiment 2 with only left-eye data. Blinks were defined as the samples 50 ms before and after the pupil data was missing for three or more consecutive measures. Saccades were shifts in eye position with a velocity above 30°/s and an acceleration above 8000°/${s}^{2}.$ Fixations were samples that are neither saccades nor blinks. Preprocessed data from DataViewer was further analyzed using custom-written MATLAB^54^ scripts. We selected endpoint fixations of the primary and secondary (first corrective) saccades from each trial to calculate a subject’s first and second estimate of their visually or memory-guided response to the target location. Primary and secondary saccades were the first and the second saccade after the fixation offset at the end of the delay period (MGS) or after the target onset (VGS) with its startpoint position 2° within the center fixation and its endpoint position 5° (MGS) or 3° (VGS) within the target position. For both the MGS and VGS experiments, we calculated errors in the primary and secondary saccade endpoint position from the target position.

#### VSWM error calculation

VSWM errors were calculated as the euclidean distance in degrees of visual angle between the saccade endpoint and the target location. Systematic and unsystematic errors were separately calculated for each task condition (delay and eccentricity). Systematic error was the distance between the target position and the mean saccade endpoint position across all the trials of each task condition:

$$Systematic\,Error = \sqrt{(X-\overline{X})^{2}+(Y-\overline{Y})^{2}}$$

$X$ and $Y$ are the horizontal and vertical positions of the target, $\overline{X}$ and $\overline{Y}$ represent the horizontal and vertical positions of the mean saccade position. Unsystematic error was the average trial-by-trial deviation of saccade endpoints from the mean across all the trials of each task condition:

$$Unsystematic~Error~ = ~\frac{{\mathop \sum \nolimits_{{i~ = ~1}}^{N} \sqrt {\left( {x_{i} - \bar{X}} \right)^{2} + \left( {y_{i} - \bar{Y}} \right)^{2} } }}{N}$$

where $x_{i}$ and $y_{i}$ are the horizontal and vertical positions of the endpoint saccade for trial $i$, $N$ is the number of trials in the task condition.

We removed trials that met one of the following criteria: (1) Eye positions during any time of the stimulus or the delay period exceeded a 2°-window from the central fixation (for the MGS); (2) Reaction time of the primary saccades was shorter than 80 ms or longer than 800 ms; (3) No saccades during the probe period met the selection criteria of primary or secondary saccades; (4) Bivariate error was more than three median absolute deviations away from the median errors of all trials. On average, we removed 8.8% $\pm$ 8.0% (MGS) and 1.7% $\pm$ 1.5% (VGS) of the trials in Experiment 1. We removed 5.1% $\pm$ 2.3% (MGS, main dataset), 4.7% $\pm$ 2.1% (VGS, main dataset), 5.1% $\pm$ 1.6% (MGS, replication dataset), and 5.2% $\pm$ 2.4% (VGS, replication dataset) of the trials in Experiment 2.

#### Statistical testing

We performed multiple two-way repeated-measures ANOVAs on the primary and secondary saccades errors with task, eccentricity, and delay as within-subject factors. Mauchly’s test of sphericity was used to examine if data violates the assumption of sphericity, followed by the Greenhouse–Geisser correction. For significant main effects of the ANOVA, we performed post-hoc polynomial contrasts and/or multiple comparisons with Bonferroni correction. We conducted simple main effects analyses for any higher-order interaction effects, followed by polynomial contrasts, multiple comparisons, and interaction contrasts analyses with Bonferroni correction.

In Experiment 2, we also analyzed data at the individual level by regressing the MGS errors on eccentricity, delay duration, and their product for each subject. We calculated the 95% CI of each coefficient and reported the results in the supplementary materials (Supplementary Table S2; Fig. S4).

### Computational simulation

Neural representation of VSWM was simulated by a 1D continuous bump attractor model^52^. This model consists of the same number of excitatory and inhibitory neurons, each of which is characterized by a firing-rate model and labeled by an angle θ that represents its receptive field. Neurons were mutually connected; connectivity between neurons was characterized by four matrices ($W_{{EE}} ,W_{{II}} ,W_{{IE}} ,W_{{EI}}$). Connectivity within the excitatory neuron population ($W_{{EE}}$) was modeled by a circular gaussian function of the distance between the angle of neuron i and that of neuron j ($\theta _{i}$- $\theta _{j}$). All other connectivity matrices are homogeneous.

We simulated the firing rate of N excitatory and inhibitory neurons during an MGS task, from which behavioral response was decoded at the end of the delay period using a population vector decoder. The MGS task consisted of a 2-s pre-stimulus period and 0.2-s stimulus presentation, followed by a delay period with varying duration (0.5, 1, 1.5, 2, 3, 4, 5 s) as in Experiment 2. Separate simulations were run for different eccentricities and delay durations. The number of neurons in the model, N, decreased with increasing eccentricity to simulate the effect of central to peripheral visual representation in the early visual cortex. N at each eccentricity was calculated based on the cortical magnification factor estimated by the following Eq. ^55,86^

$$M^{{ - 1}} = M_{0}^{{ - 1}} \cdot \left( {1 + E \cdot E_{2}^{{ - 1}} } \right),\,M_{0} = 22.5,\,E_{2} = 0.785$$

Values of $M_{0}$ and $E_{2}$ were estimated by retinotopic mapping of the human visual cortex^55,86^. N was set at 200 when M was 1. The final model had 933 neurons for the small eccentricity (3° of visual angle), 402 neurons for the medium eccentricity (8° of visual angle), and 256 neurons for the large eccentricity (13° of visual angle).

For each combination of eccentricity and delay, we simulated 10,000 independent trials. We then calculated systematic errors and unsystematic errors from all the simulated trials and reported the results in the supplementary material (Supplementary Fig. S5).

## Supplementary Information

Supplementary Information 1.
