# Supplementary material for: Errors in visuospatial working memory across space and time
Source: Sci Rep. 2021 Jul 14;11:14449. doi: 10.1038/s41598-021-93858-6 (PMC8280190; doi:10.1038/s41598-021-93858-6)
Supplement: Supplementary file 1 — Supplementary Information 1. [file 41598_2021_93858_MOESM1_ESM.pdf]

Supplementary Materials

Errors in Visuospatial Working Memory Across Space and Time

Linjing Jiang*, Hoi-Chung Leung*

Integrative Neuroscience Program, Department of Psychology, Stony Brook University, Stony Brook, NY 11794

*linjing.jiang@stonybrook.edu, hoi-chung.leung@stonybrook.edu

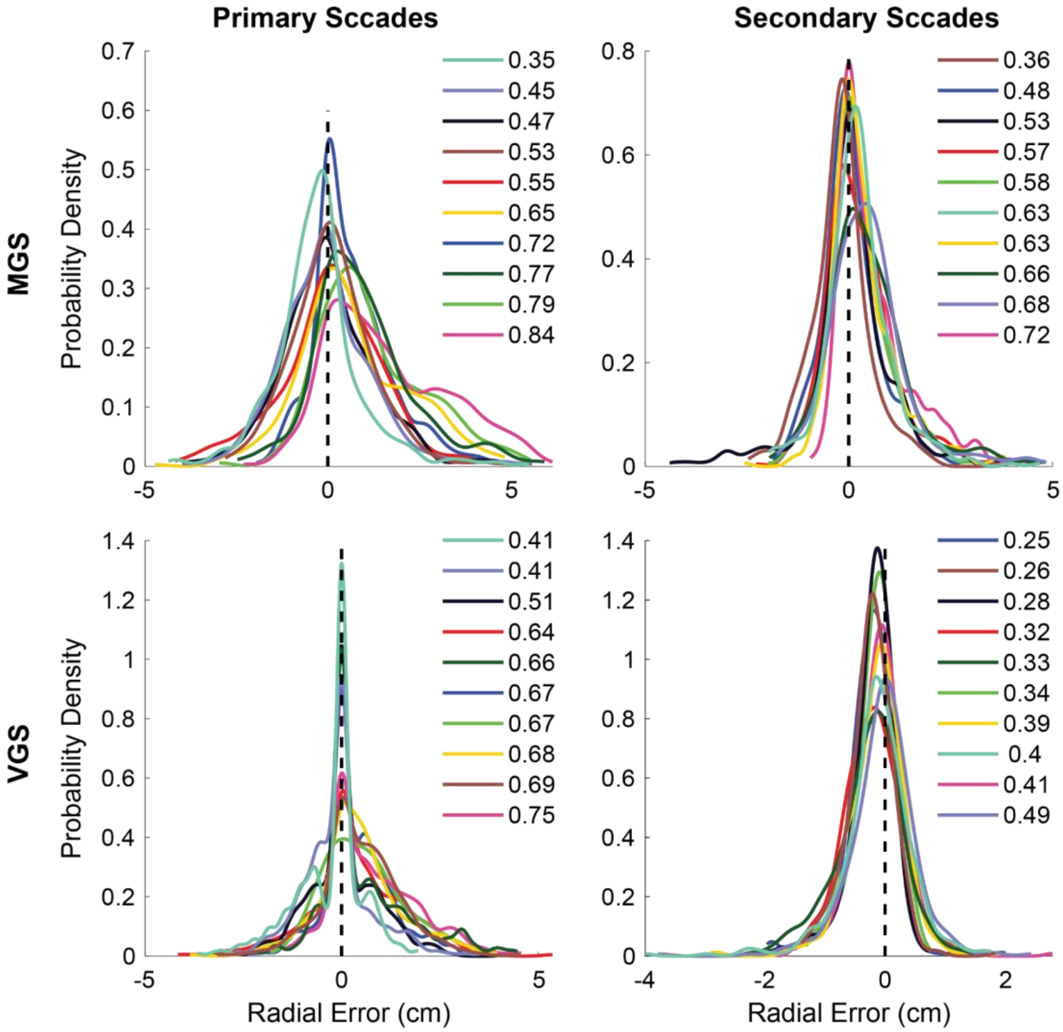

Figure S1. The probability density distribution of the radial errors showed fovea bias across individual subjects in Experiment 1. Radial errors were the target radius minus the saccadic response radius (in cm). The vertical dashed lines denote zero radial error. The right (left) side of the vertical line represents positive (negative) radial errors, indicating that the response is hypometric (hypermetric) relative to the target. Curve lines show the probability density distributions of each subject. The legend shows the cumulative probability of each subject's hypometric errors (fovea bias) ascending in order. Paired t-test results showed that the primary saccades were hypometric for both the MGS ($t(9) = 2.19$, $p = .06$) and VGS task ($t(9) = 2.56$, $p = .03$), whereas secondary saccades were hypometric for the MGS task ($t(9) = 2.86$, $p = .02$) but

hypermetric ($t(9) = -6.37$, $p = .02$) for the VGS task, indicating a fovea bias in the systematic errors of the MGS.

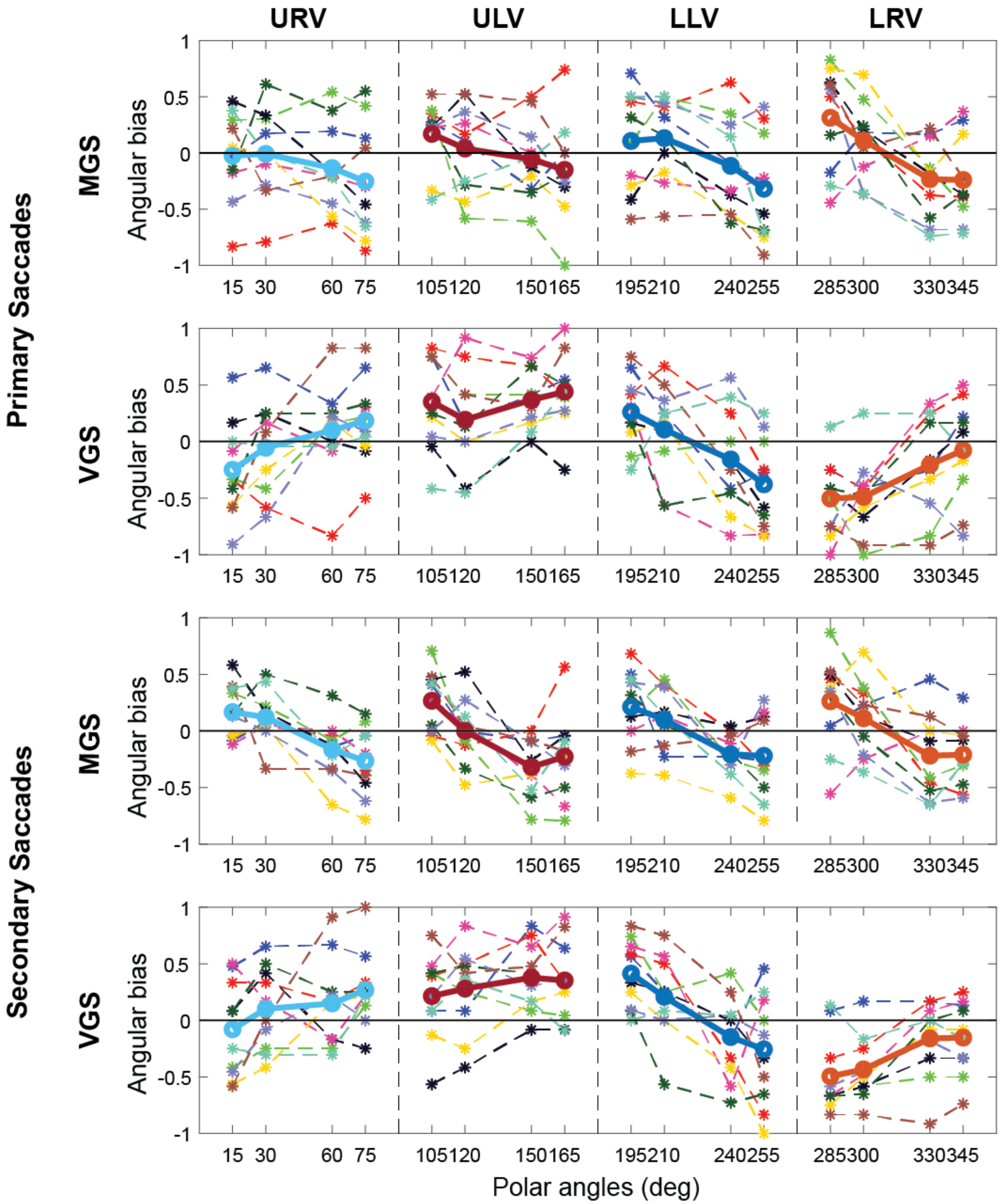

Figure S2. Angular errors (in polar angles) of individual subjects showed quadrant bias in Experiment 1. We calculated angular biases as the proportion of trials with a response counterclockwise relative to the target minus the proportion of trials with a clockwise response. Dashed lines show individual subjects' angular biases over the four polar angles within a quadrant, while solid lines show the group averages. Quadrant bias seemed evident exclusively for the MGS responses. As the target's polar angle increased within each quadrant, angular bias appeared to decrease from positive to negative, crossing the x-axis at about the center angle of the quadrant. In other words, saccade endpoints landed counterclockwise (clockwise) relative to the targets located clockwise (counterclockwise) to the center of the quadrant, indicating an attraction of saccade endpoints toward the quadrant center. Such patterns were not evident in the VGS responses except for the third quadrant (lower left visual field). URV: upper right visual field; ULV: upper left visual field; LLV: lower left visual field; LRV: lower right visual field.

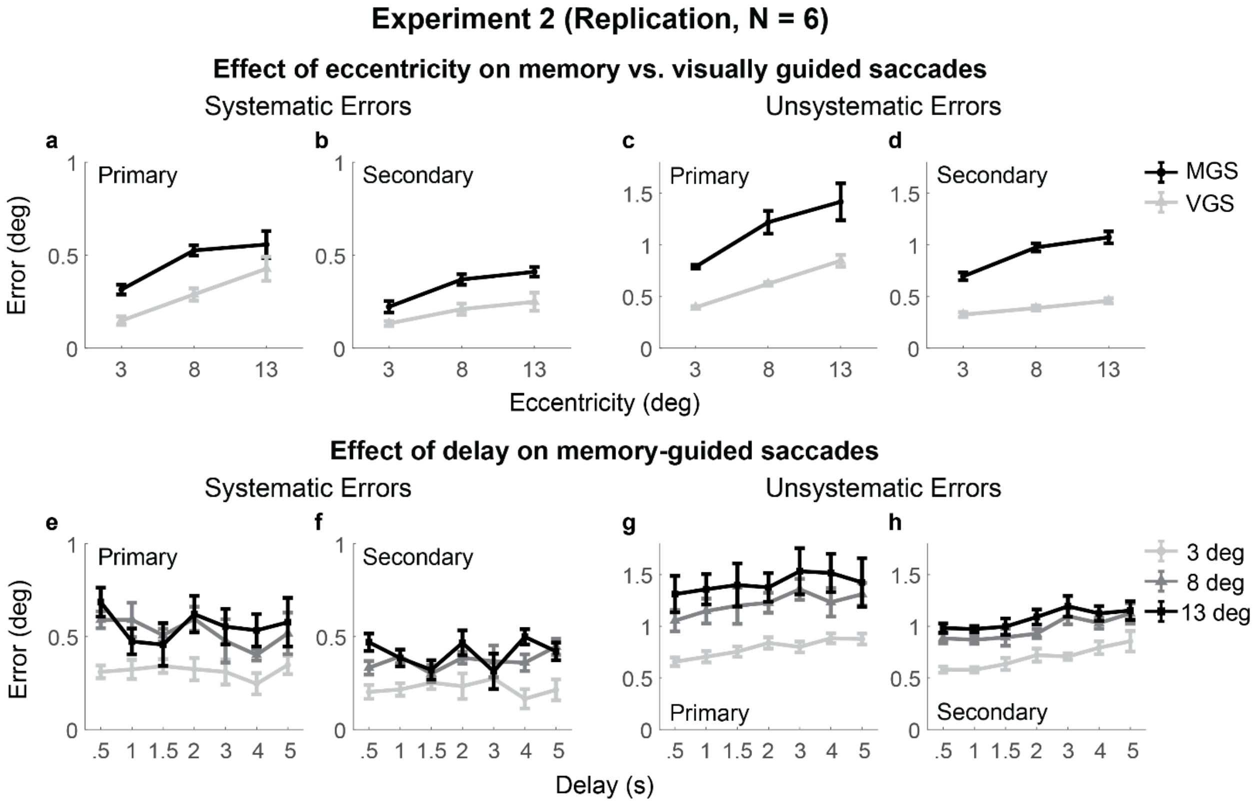

Figure S3. Saccade endpoint errors across tasks, eccentricities, and delays in the replication dataset of Experiment 2 (six out of nine subjects). The top row shows errors in the MGS (black) and VGS (light gray) across different target eccentricities. The bottom row shows errors in the MGS across different delay intervals for each target eccentricity (3°, light gray; 8°, gray; 13°, black). The replication dataset showed MGS patterns similar to the main dataset's (see main text, Fig. 5), with a more robust main effect of delay on the unsystematic errors of the primary saccades (Supplementary Table S1B). It suggests some minor practice differences between the two datasets: An extensive practice seemed to reduce the unsystematic errors for short delays (comparing Supplementary Fig. S3g with Fig. 5g).

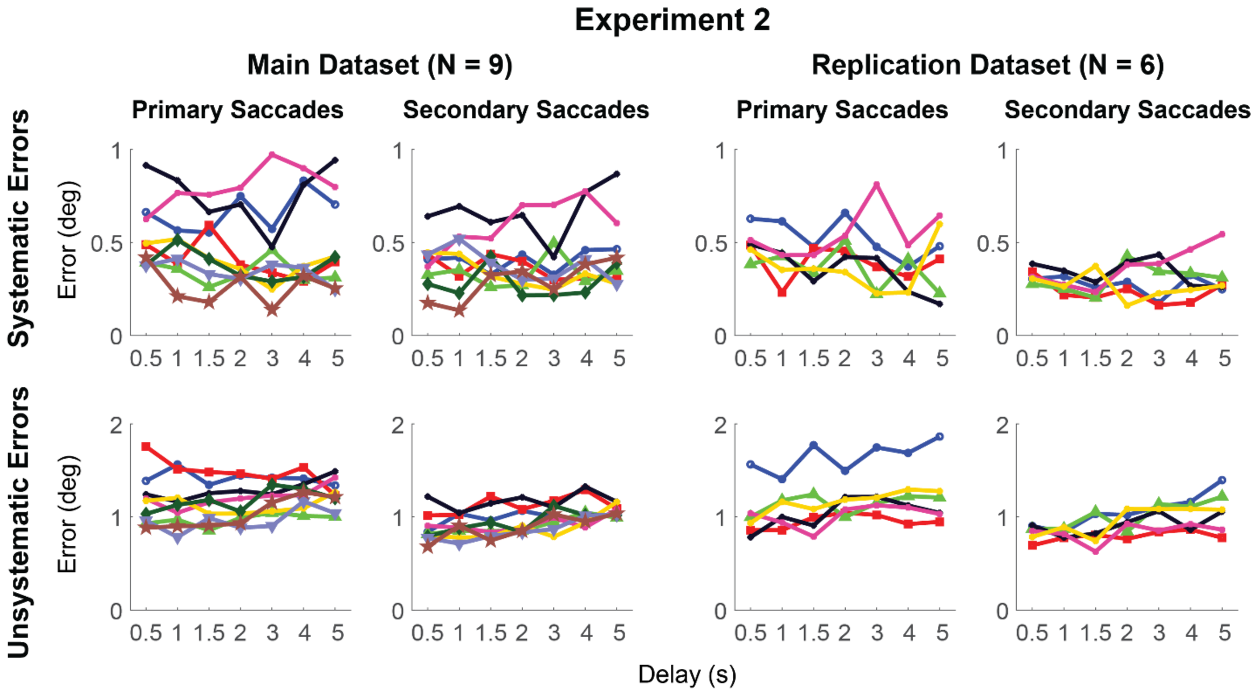

Figure S4. Individual subjects' error accumulation over delays during the MGS task in Experiment 2. Different colors represent the systematic and unsystematic errors from each subject averaged across eccentricities. The individual-level analysis was congruent with the group-level analysis (Fig. 5; Supplementary Fig. S3): The systematic errors varied randomly, whereas the unsystematic errors increased from 0.5-s to 5-s delay intervals, despite some individual differences.

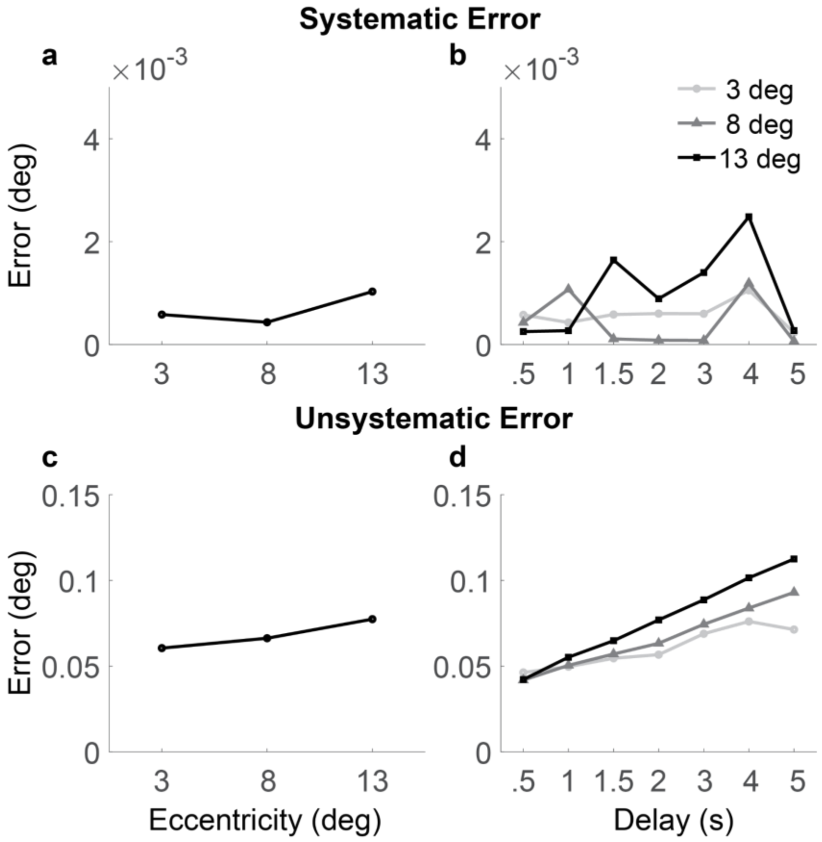

Figure S5. Saccade endpoint errors across eccentricities and delays simulated in the bump attractor model. (**a**) Simulated systematic errors at three eccentricities, averaged across delays. (**b**) Simulated systematic errors over delays at the eccentricity of 3° (933 neurons; light gray), 8° (402 neurons; gray), and 13° (256 neurons; black) of visual angle. Respective results for the unsystematic errors are shown in (**c**) and (**d**). Simulated unsystematic errors, not systematic errors, increased with increasing eccentricity and longer delays with a substantial delay by eccentricity interaction. See similar findings in Fig. 5 in a paper by Compte and colleagues[51].

A. Effects of task, eccentricity, and their interaction

	Primary, Systematic	Primary, Unsystematic	Secondary, Systematic	Secondary, Unsystematic
Effect of Task	$F(1,5) = 98.19$, $p < .001$, $\eta_p^2 = .95$	$F(1,5) = 38.35$, $p = .002$, $\eta_p^2 = .89$	$F(1,5) = 13.86$, $p = .014$, $\eta_p^2 = .73$	$F(1,5) = 78.29$, $p < .001$, $\eta_p^2 = .94$
Effect of Eccentricity	$F(2,10) = 10.99$, $p < .017^a$, $\eta_p^2 = .69$	$F(2,10) = 19.26$, $p = .005^a$, $\eta_p^2 = .79$	$F(2,10) = 13.51$, $p = .006$, $\eta_p^2 = .73$	$F(2,10) = 50.63$, $p < .001$, $\eta_p^2 = .91$
Task* Eccentricity	$F(2,10) = 1.10$, $p = .36$, $\eta_p^2 = .18$	$F(2,10) = 2.06$, $p = .20$, $\eta_p^2 = .29$	$F(2,10) = 0.82$, $p = .44$, $\eta_p^2 = .14$	$F(2,10) = 14.06$, $p = .005$, $\eta_p^2 = .74$

B. Effects of delay, eccentricity and their interaction in MGS

	Primary, Systematic	Primary, Unsystematic	Secondary, Systematic	Secondary, Unsystematic
Effect of Eccentricity	$F(2,10) = 8.50$, $p = .007$, $\eta_p^2 = .63$	$F(2,10) = 10.11$, $p = .004$, $\eta_p^2 = .67$	$F(2,10) = 10.56$, $p = .003$, $\eta_p^2 = .68$	$F(2,10) = 31.05$, $p < .001$, $\eta_p^2 = .86$
Effect of Delay	$F(6,30) = 1.06$, $p = .41$, $\eta_p^2 = .18$	$F(6,30) = 3.42$, $p = .011$, $\eta_p^2 = .41$	$F(6,30) = 0.71$, $p = .64$, $\eta_p^2 = .12$	$F(6,30) = 5.72$, $p < .001$, $\eta_p^2 = .53$
Eccentricity* Delay	$F(12,60) = 0.60$, $p = .83$, $\eta_p^2 = .11$	$F(12,60) = 0.52$, $p = .89$, $\eta_p^2 = .10$	$F(12,60) = 1.23$, $p = .28$, $\eta_p^2 = .20$	$F(12,60) = 0.57$, $p = .86$, $\eta_p^2 = .10$

Table S1. Repeated-measures analyses of variance on VSWM errors from the second dataset of Experiment 2. "a" indicates p-value corrected by the Greenhouse-Geisser method due to violation of sphericity.

	Subject	Constant	Eccentricity	Delay	Eccentricity x Delay
Primary, Systematic	1	[-0.053,0.65]	[-0.002,0.077]	[-0.14,0.11]	[-0.007,0.020]
	2	[-0.20,0.35]	**[.028, .090]**	[-0.092, 0.10]	[-.017,0.005]
	3	[-0.18,0.52]	[-0.013,0.065]	[-0.13,0.12]	[-0.014,0.013]
	4	**[0.17,1.09]**	[-0.034,0.069]	[-0.24,0.079]	[-0.007,0.029]
	5	**[0.26,0.79]**	[-0.005,0.054]	[-0.077,0.11]	[-0.007,0.014]
	6	[-0.16,0.59]	[-0.009,0.075]	[-0.11,0.15]	[-0.019,0.010]
	7	[-0.038,0.41]	**[0.008,0.058]**	[-0.018,0.14]	[-0.017,0.0003]
	8	[-0.054,0.52]	[-0.007,0.056]	[-0.11,0.085]	[-0.012,0.011]
	9	**[0.15,0.60]**	[-0.034,0.016]	[-0.040,0.12]	[-0.014,0.004]
	1*	[-0.043,0.69]	**[0.005,0.086]**	[-0.22,0.039]	[-0.009,0.020]
	2*	[-0.033,0.55]	[-0.011,0.054]	[-0.11,0.090]	[-0.011,0.011]
	3*	[-0.13,0.62]	[-0.010,0.074]	[-0.14,0.12]	[-0.017,0.012]
	4*	**[0.17,0.74]**	[-0.022,0.041]	[-0.15,0.045]	[-0.011,0.011]
	5*	[-0.13,0.53]	[-0.003,0.070]	[-0.025,0.20]	[-0.018,0.008]
	6*	[-0.11,0.79]	[-0.046,0.054]	[-0.16,0.16]	[-0.016,0.019]
Primary, Unsystematic	1	**[0.040,0.80]**	**[0.087,0.17]**	[-0.12,0.15]	[-0.020,0.010]
	2	[-0.13,1.34]	**[0.043,0.21]**	[-0.19,0.32]	[-0.045,0.012]
	3	**[0.40,0.90]**	**[0.003,0.058]**	[-0.069,0.10]	[-0.008,0.011]

	4	**[0.94,1.46]**	[-0.036,0.021]	[-0.14,0.038]	**[0.003,0.023]**
	5	**[0.63,1.15]**	[-0.007,0.050]	[-0.067,0.11]	[-0.006,0.014]
	6	[0.26,1.02]	**[0.013,0.097]**	[-0.095,0.17]	[-0.019,0.011]
	7	**[0.005,0.90]**	**[0.024,0.12]**	[-0.063,0.25]	[-0.023,0.012]
	8	**[0.20,0.80]**	**[0.011,0.078]**	[-0.035,0.17]	[-0.015,0.009]
	9	**[0.30,0.86]**	[-0.003,0.059]	[-0.052,0.14]	[-0.005,0.017]
	1*	**[0.004,0.73]**	**[0.098,0.18]**	[-0.13,0.12]	[-0.005,0.023]
	2*	**[0.25,0.74]**	**[0.024,0.078]**	[-0.059,0.11]	[-0.011,0.008]
	3*	[-0.013,0.73]	**[0.043,0.13]**	[-0.003,0.25]	[-0.026,0.003]
	4*	**[0.41,1.07]**	[-0.017,0.057]	[-0.12,0.11]	[-0.006,0.020]
	5*	**[0.13,0.67]**	**[0.034,0.095]**	**[0.030,0.22]**	[-0.022,-0.0009]
	6*	**[0.032,1.05]**	[-0.002,0.079]	[-0.074,0.18]	[-0.013,0.015]
Secondary, Systematic	1	**[0.12,0.65]**	[-0.027,0.032]	[-0.17,0.014]	**[0.001,0.022]**
	2	[-0.005,0.45]	**[0.003,0.054]**	[-0.13,0.034]	[-0.006,0.012]
	3	[-0.096,0.30]	**[0.008,0.052]**	[-0.056,0.08]	[-0.008,0.008]
	4	[-0.11,0.66]	[-0.002,0.08]	[-0.12,0.14]	[-0.012,0.018]
	5	**[0.052,0.53]**	[-0.004,0.049]	[-0.026,0.14]	[-0.009,0.010]
	6	[-0.011,0.50]	[-0.008,0.050]	[-0.086,0.093]	[-0.013,0.007]
	7	[-0.15,0.51]	[-0.020,0.054]	[-0.079,0.15]	[-0.016,0.010]
	8	**[0.018,0.58]**	[-0.010,0.053]	[-0.14,0.057]	[-0.010,0.012]

	9	**[0.056,0.50]**	[-0.036,0.013]	[-0.057,0.098]	[-0.004,0.013]
	1*	[-0.075,0.37]	**[0.001,0.051]**	[-0.11,0.043]	[-0.005,0.012]
	2*	[-0.12,0.23]	**[0.008,0.047]**	[-0.063,0.060]	[-0.008,0.006]
	3*	[-0.12, 0.41]	[-0.008,0.052]	[-0.087,0.10]	[-0.009,0.011]
	4*	**[0.074,0.62]**	[-0.023,0.038]	[-0.14,0.54]	[-0.008,0.013]
	5*	[-0.12,0.43]	[-0.020,0.040]	[-0.004,0.19]	[-0.015,0.007]
	6*	[-0.003,0.56]	[-0.026,0.037]	[-0.13,0.069]	[-0.007,0.015]
Secondary, Unsystematic	1	**[0.12,0.80]**	**[0.021,0.097]**	[-0.11,0.12]	[-0.013,0.014]
	2	**[0.26,0.92]**	**[0.019,0.094]**	[-0.074,0.16]	[-0.016,0.010]
	3	**[0.31,0.76]**	**[0.003,0.054]**	[-0.057,0.10]	[-0.006,0.012]
	4	**[0.45,10.01]**	**[0.014,0.077]**	[-0.048,0.15]	[-0.015,0.007]
	5	**[0.32,0.72]**	**[0.015,0.059]**	[-0.044,0.093]	[-0.006,0.009]
	6	**[0.28,0.79]**	[-0.009,0.048]	[-0.069,0.11]	[-0.003,0.016]
	7	**[0.024,0.63]**	**[0.025,0.092]**	[-0.005,0.21]	[-0.018,0.006]
	8	**[0.042,0.44]**	**[0.033,0.078]**	**[0.052,0.19]**	[-0.15,0.001]
	9	**[0.15,0.66]**	**[0.011,0.067]**	[-0.008,0.17]	[-0.012,0.008]
	1*	**[0.025,0.57]**	**[0.031,0.092]**	[-0.008,0.18]	[-0.009,0.013]
	2*	**[0.33,0.67]**	**[0.010,0.048]**	[-0.051,0.069]	[-0.006,0.008]
	3*	**[0.086,0.73]**	**[0.013,0.085]**	**[0.026,0.25]**	[-0.020,0.005]
	4*	**[0.31,0.71]**	**[0.015,0.059]**	**[0.011,0.15]**	[-0.013,0.003]

5*	**[0.016,0.72]**	**[0.010,0.072]**	[-0.049,0.15]	[-0.013,0.008]
6*	[0.20,0.83]	[-0.0009,0.069]	[-0.060,0.16]	[-0.010,0.014]

Table S2. 95% Confidence Intervals (CI) of the coefficients in a linear regression of MGS errors against eccentricity, delay, and their interaction in Experiment 2. "*" indicates the second dataset collected for Experiment 2 from six of the nine subjects. Results in bold indicate a 95% CI that does not include 0.
